# Mejora del protocolo de cribado de diabetes gestacional: estudio de validación diagnóstica

**DOI:** 10.1515/almed-2020-0118

**Published:** 2021-02-11

**Authors:** Miguel Calero Rojas, Aurora Jurado Roger, Marta Gutiérrez Grúa, Lourdes de la Peña Carretero, Victoria Romero Sotomayor, Javier López Braos, Federico Izquierdo Carrasco, Luis Herrero Tabanera, Carmen Moreno Aguilar

**Affiliations:** Obstetrics and Gynaecology Unit, Hospital Infanta Margarita, Cabra, España; Immunlogy and Allergy Unit, Hospital Universitario Reina Sofía- IMIBIC, Córdoba, España; Laboratory Unit, Hospital Infanta Margarita, Cabra, España; Operation Control Unit, Hospital Infanta Margarita, Cabra, España; Departamento de Inmunología y Alergia, Hospital Universitario Reina Sofía, Avda. Menéndez Pidal s/n, 14004, Córdoba, España

**Keywords:** eventos adversos, diabetes gestacional, test de O’Sullivan, hemoglobina glIcosilada, cribado

## Abstract

**Objetivos:**

El objetivo de este trabajo es evaluar la validez diagnóstica de dos métodos de cribado de diabetes mellitus gestacional (DMG).

**Métodos:**

Se realizó un estudio prospectivo de validación diagnóstica en 2007 embarazadas. Las participantes fueron asignadas al grupo de alto o bajo riesgo, dependiendo de los factores de riesgo que presentaran. Se realizó una prueba de HbA_1c_ simultáneamente al protocolo estándar basado en el test de O’Sullivan (TOS). Posteriormente, se aplicó un algoritmo que combinaba los resultados de ambos biomarcadores.

**Resultados:**

En el grupo de bajo riesgo, el TOS mostró un área bajo la curva mayor (AUC 0,953; IC95% 0,915–0,992) que la prueba de HbA_1c_ (0,688; IC 95% 0,541–0,834). El mejor punto de corte del TOS, 153,5 mg/dL (8,52 mmol/L), mostró mayor validez diagnóstica que el de la HbA_1c_, 28 mmol/mol (4,75%) y que el algoritmo basado en ambas pruebas. En el grupo de alto riesgo, el TOS mostró mejor rendimiento diagnóstico que la HbA_1c_ y el algoritmo. Los puntos de corte óptimos para el TOS fueron mayores que los recomendados en los protocolos actuales. Semana 13: TOS AUC 0,882 (IC 95% 0,843–0,921), HbA_1c_ AUC 0,624 (IC 95% 0,562–0,686), punto de corte para el TOS 140,5 mg/dL (7,8 mmol/L), punto de corte para la HbA_1c_ 33 mmol/mol (5,15%). Semana 24: TOS AUC 0,944 (IC 95% 0,925–0,962), HbA_1c_ AUC 0,642 (IC 95% 0,575–0,709), punto de corte para el TOS, 145,5 mg/dL (8,08 mmol/L), punto de corte para la HbA_1c_ 29 mmol/mol (4,85%).

**Conclusiones:**

El método para el cribado de DMG basado en el test de O’Sullivan con valores límite superiores a los recomendados fue el que mostró mejor validez diagnóstica. Si se hubieran aplicado estos umbrales, se habría evitado la prueba de sobrecarga oral de glucosa con 100 g al 55,6% y al 13,7% de las gestantes de bajo y alto riesgo.

## Introducción

La diabetes mellitus gestacional (DMG) se ha definido tradicionalmente como cualquier grado de intolerancia a la glucosa que aparece o es identificada por primera vez durante el embarazo [1]. Esta definición ha sido modificada recientemente por la Organización Mundial de la Salud [2] y la Asociación Americana de Diabetes [3], pasando a aplicar el término DMG a aquellos casos diagnosticados en el segundo o tercer trimestre de embarazo, que no sean diabetes de tipo 1 o 2. Sin embargo, estos nuevos criterios no han sido aceptados universalmente, y la definición y el diagnóstico de la misma sigue siendo objeto de un intenso debate [4], [5], [6], [7].

De acuerdo con la definición clásica de DMG, su prevalencia oscila entre el 2% y el 17% [7], [ 8]. En España, la probabilidad de desarrollar DMG es del 8,8% [9].

El Grupo Español de Diabetes y Embarazo (GEDE) clasifica a las embarazadas en dos grupos: alto riesgo, y riesgo bajo/moderado. La DMG está relacionada con los siguientes factores de riesgo: edad (>35 años), obesidad (IMC >30 kg/m^2^), antecedentes de DMG, familiar de primer grado con DM, y antecedentes de macrosomía [10].

El método validado en España para el diagnóstico de DMG consta de dos pasos: el cribado con el test de O’Sullivan (TOS) y la prueba de sobrecarga oral de glucosa (SOG). El TOS se realiza en la semana 24^a^ de gestación (semana 13^a^, si existe algún factor de riesgo). Si el TOS es positivo (≥140 mg/dL; ≥7,77 mmol/L), se realiza la SOG. El TOS consiste en la administración oral de 50 g de glucosa y la medición de glucemia al cabo de una hora. La SOG consiste en la administración de 100 g de glucosa, que se mide antes de la administración y transcurridas una, dos y tres horas [10], [ 11]. Las gestantes suelen mostrar mala tolerancia a estas pruebas, que suelen provocarles vómitos e impiden que la prueba llegue a término. Otras limitaciones de estos métodos son la duración y la glucólisis preanalítica en muestras de glucosa en plasma, que pueden llevar al infradiagnóstico de la DMG [4], [6], [12], [13], [14], [15], [16].

En 2009, el Comité Internacional de Expertos en Diabetes recomendó la prueba de la hemoglobina A glicosilada (HbA_1c_) como la prueba de elección para el manejo crónico de la diabetes [16]. La prueba de la HbA_1c_ ofrece varias ventajas frente a las pruebas basadas en los niveles de glucosa en plasma, como son la estandarización, una mejor correlación con la incidencia de eventos adversos a largo plazo, una menor incidencia de errores preanalíticos, no precisar la toma secuencial de varias muestras y un menor impacto causado por las perturbaciones agudas en los niveles de glucosa. No obstante, la precisión diagnóstica de la HbA_1c_ en el diagnóstico de la diabetes durante el embarazo, un periodo en el que se producen cambios en el recambio de eritrocitos, podría disminuir, por lo que se recomienda realizar pruebas basadas en la medición de glucosa [7], [ 17].

Diversos artículos han demostrado que la prueba de la HbA_1c_ podría ser útil en el diagnóstico de DM en individuos con alto riesgo [18], [ 19]. Sin embargo, aún no se han realizado suficientes estudios que validen el uso de la HbA_1c_ en el diagnóstico de la DMG [20], [21], [22], [23], [24] y su utilidad sigue siendo objeto de controversia [25].

De este modo, el propósito de este estudio es evaluar la validez diagnóstica de dos nuevas estrategias, que causen menos molestias a las embarazadas, para el cribado de DMG.

## Materiales y métodos

Este estudio prospectivo se realizó en un hospital terciario (Hospital Infanta Margarita). Se incluyó a todas las embarazadas tratadas en el hospital y en su área de referencia (Sur de Córdoba) durante un periodo de tres años. La investigación se realizó de conformidad con la legislación nacional, las políticas institucionales, y de acuerdo con los principios de la Declaración de Helsinki. Así mismo, este estudio fue aprobado por el Comité de Ética del Hospital Reina Sofía, Córdoba (España). Este estudio fue financiado por una ayuda del Instituto Nacional de Salud Carlos III (ISCIII) (PI11 01064).

Asumiendo una prevalencia del 10%, una sensibilidad del 85%, una especifidad del 85%, y un porcentaje permitido de errores de tipo II del 5%, con un nivel de significación del 95%, con el programa Epidat 4.1 (Conselleria de Sanidade Galega) se calculó una muestra de 1.970 participantes.

Se incluyó a todas las gestantes atendidas en la Unidad de Obstetricia entre septiembre de 2011 y septiembre de 2014 que aceptaron participar en el estudio. Todas las participantes firmaron un consentimiento informado. Se excluyó a las mujeres con diabetes pregestacional, hemoglobinopatías o cualquier situación asociada con un mayor recambio de glóbulos rojos (anemia, transfusión).

La muestra inicial estaba compuesta por 2.270 mujeres. Se excluyó a 221 por diversas razones: pérdida durante el seguimiento (134), aborto espontáneo (39), seguimiento en un centro privado (31) y datos demográficos incompletos (17). Además, se excluyó del análisis final a 42 participantes, debido a que recibieron una transfusión (35), presentaron diabetes pregestacional (3), hemoglobinopatías (2) o abandonaron el estudio. La muestra final incluida en el análisis estadístico estuvo compuesta por un total de 2,007 mujeres.

A las participantes se les realizó un examen físico y se les entregó un cuestionario para identificar factores de riesgo de DMG: edad (>35 años), obesidad (IMC >30 kg/m^2^), antecedentes de DMG, familiar de primer con DM, antecedentes de macrosomía y origen étnico con elevada prevalencia de DMG (africanas, latinoamericanas o asiáticas).

Se realizó el TOS y una prueba de HbA_1c_ entre la semana 24^a^ y la semana 26^a^ de embarazo (GCT-s24; HbA_1c_-s24). Si los resultados del TOS eran positivos (≥140 mg/dL; ≥7,77 mmol/L), se realizó la SOG con 100 g. Una SOG se consideró positiva si ≥105 mg/dL (≥5,83 mmol/L) (basal), ≥190 mg/dL (≥10,55 mmol/L) (1 h), ≥165 mg/dL (≥9,16 mmol/L) (2 h), ≥145 mg/dL (≥8,05 mmol/L) (3 h) [6], [ 7]. El diagnóstico de DMG se confirmó si se cumplía alguno de los siguientes criterios: nivel de glucosa en plasma ≥126 mg/dL (≥6,99 mmol/L) (medido dos veces), glucemia aleatoria ≥200 mg/dL (≥11,1 mg/L) y dos o más puntos alterados en la SOG (según los criterios del GEDE [10] y del Grupo Nacional de Datos sobre la Diabetes [11]). Las mujeres que presentaron factores de riesgo de DMG fueron evaluadas en dos ocasiones siguiendo el mismo esquema: en la semana 13^a^ de gestación (GCT-s13; HbA_1c_ -s13) y entre la semana 24^a^ y 26^a^

Dependiendo de la epidemiología, historia clínica y los resultados analíticos, los obstetras, que desconocían los resultados de la HbA_1c_ asignaron a las participantes a diferentes grupos.

El análisis clínico de las pruebas de glucosa y de HbA_1c_ fue realizado por diferentes técnicos de laboratorio y diferentes facultativos, que desconocían respectivamente el resultado de la prueba alternativa, así como el grupo al que pertenecían las participantes.

**Procedimientos analíticos** (véase el Material Suplementario).

**Análisis estadístico** (véase el Material Suplementario).

**Estrategias para el cribado de la DMG** (véase Material Suplementario).

## Resultados

La distribución de frecuencias de las variables continuas no presentó una distribución normal en ninguna de las cohortes (población total, gestantes con o sin factores de riesgo). En la Tabla 1 se detallan los resultados del análisis descriptivo.

**Tabla 1:** Distribución de las variables clínicas de la población del estudio.

Variables clínicas	Todas las gestantes (n=2007)	Grupo de bajo riesgo (n=1054)	Grupo de alto riesgo (n=953)
	Mediana (IR^a^)	Mediana (RI)	Mediana (RI)
Edad, años	31 (28–35)	29 (26–32)	34 (31–37)
TOS-s13^b^, mg/dL	na^c^	na	109 (89–131)
TOS-s13, mmol/L	na	na	6,05 (4,94–7,27)
HbA_1c_-s13^d^, mmol/mol	na	na	31,1 (29–33,3)
HbA_1c_-s13, %	na	na	5 (4,8–5,2)
TOS-s24^e^, mg/dL	115 (96–138)	110 (93–132)	122 (102–144)
TOS-s24, mmol/L	6,38 (5,33–7,66)	6,11 (5,16–7,33)	6,77 (5,66–7,99)
HbA_1c_-s24^f^, mmol/mol	29 (25,7–31,1)	28 (25,7–31,1)	29 (26,8–32,2)
HbA_1c_-s24, %	4,8 (4,5–5)	4,7 (4,5–5)	4,8 (4,6–5,1)

	**%**	**%**	**%**

Prevalencia DMG^g^	5,7	1,8	10
Factores de riesgo	47,5	0	1
Edad>35 años	22,5	0	47,4
BMI^h^>30 kg/m^2^	13,7	0	28,8
Macrosomía	2,2	0	4,7
HP^i^ de DG	3,4	0	7,1
HF^j^ de diabetes	24,7	0	51,9
Origen étnico de riesgo	0,1	0	0,2

RI^a^, rango intercuartílico; TOS-s13^b^, test de O’Sullivan en la semana 13^a^; na^c^, No aplicable; HbA_1c_ -s13^d^, prueba de hemoglobina glicosilada en la semana 13^a^; TOS-s24^e^, test de O’Sullivan en la semana 24^a^; HbA_1c_ -s24^f^, prueba de hemoglobina glicosilada en la semana 24^a^; DMG^g^, Diabetes mellitus gestacional; IMC^h^, Índice de masa corporal; HP^i^, Historia personal; HF^j^, historia familiar.

### Análisis de la población

La prevalencia de la DMG en toda la población fue del 5,7%. Las medianas de las variables edad, TOS-s24 y HbA_1c_-s24 fueron significativamente superiores en las embarazadas con factores de riesgo (p<0,001). Las participantes que desarrollaron DMG tenían mayor edad y presentaban niveles superiores de TOS y HbA_1c_ (p<0,001) en la semana 24^a^. La presencia de factores de riesgo, especialmente la edad superior a los 35 años, un IMC >30 kg/m^2^, antecedentes de DMG y antecedentes familiares de DM fueron significativamente más frecuentes en aquellas participantes que desarrollaron DMG, frente a las que no lo hicieron (p<0,001) (Tablas 2 y 3). Aunque 79 participantes habían nacido en otro país (20 nacionalidades distintas), solo dos de ellas no eran caucásicas.

**Tabla 2:** Comparación de variables continuas entre las gestantes que desarrollaron (casos) y no desarrollaron (controles) DMG^a^.

Población	Variables continuas	Casos: mediana (RI^b^)	Controles: mediana (RI)	VALOR p
**Todas las gestantes**	Edad, años	34 (30,75–37)	31 (28–34)	<0,001
	TOS-s24^c^, mg/dL	168 (156–187,5)	114 (96–135)	<0,001
	TOS-s24, mmol/L	9,32 (8,69–10,41)	6,33 (5,33–7,49)	
	HbA_1c_-s24^d^, mmol/mol	31 (28–36)	29 (26–31)	<0,001
	HbA_1c_-s24, %	5 (4,7–5,4)	4,8 (4,5–5)	
**Gestantes sin factores de riesgo**	Edad, años	30 (28–33)	29 (26–32)	ns^e^
	TOS-s24, mg/dL	167 (160–180)	110 (92–130)	<0,001
	TOS-s24, mmol/L	9,27 (8,88–9,99)	6,11 (5,11–7,22)	
	HbA_1c_-s24, mmol/mol	31 (29–37)	28 (26–31)	0,006
	HbA_1c_-s24,%	5 (4,77–5,5)	4,7 (4,5–5)	
**Gestantes con factores de riesgo**	Edad, años	35 (31–37)	34 (31–37)	ns
	TOS-s13^f,^ mg/dL	163 (134,5–184)	106 (89–125)	<0,001
	TOS-s13, mmol/L	9,05 (7,46–10,21)	5,88 (4,77–6,94)	
	HbA_1c_-s13^g^, mmol/mol	33 (30–36)	31 (29–33)	<0,001
	HbA_1c_-s13, %	5,2 (4,9–5,4)	5 (4,8–5,2)	
	TOS-s24, mg/dL	168 (154,7–189,7)	119,5 (100–139)	<0,001
	TOS-s24, mmol/L	9,32 (6,59–10,53)	6,63 (5,55–7,71)	
	HbA_1c_-s24, mmol/mol	31 (28–34)	29 (27–32)	<0,001
	HbA_1c_-s24, %	5 (4,7–5,3)	4,8 (4,6–5,1)	

DMG^a^, diabetes mellitus gestacional; RI^b^, Rango intercuartílico; TOS-s24^c^, test de O’Sullivan en la semana 24^a^; HbA_1c_-s24^d^, prueba de hemoglobina glicosilada en la semana 24^a^; ns^e^, no significativa; TOS-s13^f^, test de O’Sullivan en la semana 13^a^; HbA_1c_-s13^g^, prueba de hemoglobina glicosilada en la semana 13^a^.

**Tabla 3:** *Odds ratios* para cada factor de riesgo (variables discretas) para desarrollar DMG^a^.

Población	Variables discretas	OR^b^	IC^c^95%	Valor p
Todas las gestantes	Tener factores de riesgo	6,03	3,65–9,95	<0,001
	Edad >35 years	2,11	1,42–3,13	<0,001
	Macrosomía			Ns^d^
	IMC^e^>30 kg/m^2^	3,83	2,54–5,76	<0,001
	HP^f^ de DMG	13,02	7,63–22,2	<0,001
	HF^g^ de DMG	2,44	1,66–3,59	<0,001
Mujeres con factores de riesgo	Edad >35 years			ns
	Macrosomía			ns
	IMC >30 kg/m^2^	1,93	1,25–2,99	<0,001
	HP de DMG	7,32	4,23–12,65	<0,001
	HF de DMG	** **	** **	ns

DMG^a^, diabetes mellitus gestacional; OR^b^, *Odds Ratio;* IC^c^, Intervalo de confianza; ns^d^, no significativo; IMC^e^, Índice de masa corporal; HP^f^, History personal; HF^g^, Historia familiar.

La regresión logística no mostró un buen rendimiento diagnóstico, ya que, aunque el análisis clasificó correctamente al 96,9% de las participantes, solo identificó correctamente el 37,7% de los casos de DMG.

**Figura 1:**
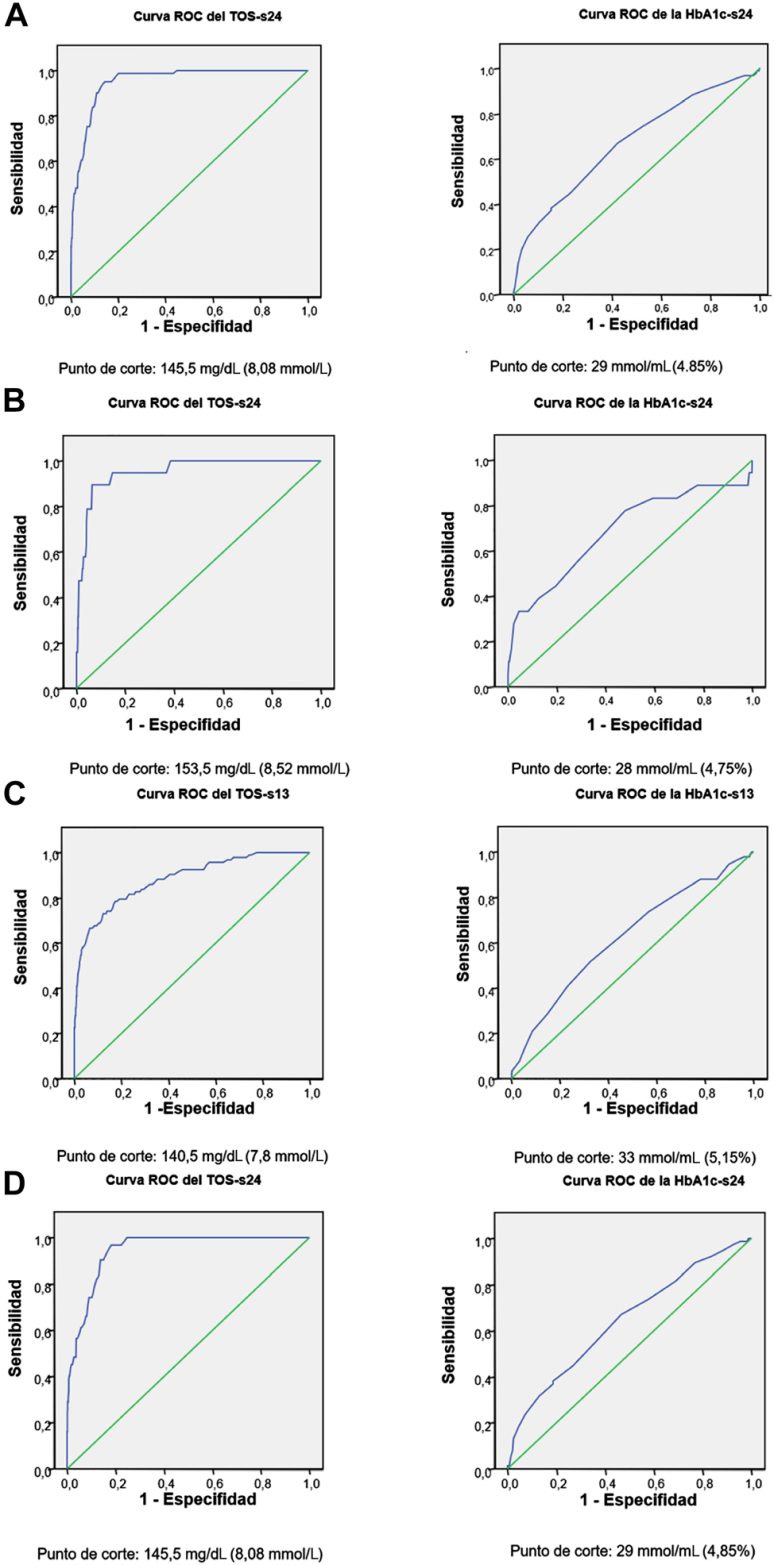
Curvas ROC que muestran la sensibilidad y especifidad del TOS^a^ y la HbA_1c_^b^ para el cribado de DMG^c^. (A) Sensibilidad y especifidad del TOS-s24d y la HbA1c-s24e en la población total. (B) Sensibilidad y especifidad del TOS-s24d y HbA1c-s24e en el grupo sin factores de riesgo. (C) Sensibilidad y especifidad del TOS-s13f y la HbA1c-s13g en el grupo con factores de riesgo. (D) Sensibilidad y especifidad del TOS-s24 y la HbA1c-s24 en el grupo con factores de riesgo. TOS^a^, test de O’Sullivan; HbA_1c_^b^, prueba de la hemoglobina glicosilada; DMG^c^, diabetes mellitus gestacional; TOS-s24^d^, test de O’Sullivan en la semana 24^a^; HbA_1c_-s24^e^, prueba de la hemoglobina glicosilada en la semana 24^a^; TOS-s13^f^, test de O’Sullivan en la semana 13^a^; HbA_1c_-s13^g^, prueba de la hemoglobina glicosilada en la semana 13^a^.

En términos de precisión diagnóstica, el TOS mostró una mayor AUC que la HbA_1c_; (0,953 vs. 0,672, respectivamente) (Figura 1A; Tabla 4). El mejor punto de corte para TOS-s24 fue 145,5 mg/dL (8,08 mmol/L) (Sensibilidad: 95,1%; Especifidad: 85,7%; VPP: 22,19%; VPN: 99,75%). Para HbA_1c_ -s24, el mejor punto de corte fue 29 mmol/mol (4,85%) (Sensibilidad: 67%; Especifidad: 57,8%; VPP: 7,6%; VPN: 97,12%) (Tabla 4).

**Tabla 4:** Estadísticos de precisión diagnóstica para cada estrategia.

Población	Prueba	Sb^a^	Sp^b^	PPV^c^	NPV^d^	AUC^e^ (95% CI^f^)
Todas las gestantes	TOS-s24^g^ ≥ 140 mg/dL (≥7,8 mmol/L)	98,8	79,9	60	99,91	0,953 (0,938–0,968)
	TOS-s24>145,4 mg/dL (>8,07 mmol/L)	95,1	85,7	22,19	99,75	0,953 (0,938–0,968)
	HbA_1c_-s24^h^ > 29 mmol/mol (>4,84%)	67	57,8	7,6	97,12	0,672 (0,612–0,731)
Gestantes sin factores de riesgo	TOS-s24≥140 mg/dL (≥7,8 mmol/L)	94,7	83,7	72	99,88	0,953 (0,915–0,992)
	TOS-s24>153,4 mg/dL (>8,51 mmol/L)	89,5	93,6	20,48	99,79	0,953 (0,915–0,992)
	HbA_1c_-s24>28 mmol/mol (>4,74%)	77,8	52,1	2,87	99,22	0,688 (0,541–0,834)
	Algoritmo combinado s24^i^	77,8	95,2	22,95	99,57	
Gestantes con factores de riesgo	TOS-s13≥140 mg/dL (≥7,8 mmol/L)	73,1	87,0	49	96,68	0,882 (0,843–0,921)
	TOS-s13^j^ > 140,4 mg/dL (>7,79 mmol/L)	73,1	87,7	39,3	96,75	0,882 (0,843–0,921)
	HbA_1c_-s13^k^ > 33 mmol/mol (>5,14%)	51,6	67,3	14,74	92,65	0,624 (0,562–0,686)
	Algoritmo combinado s13^l^	67,8	89,1	40,39	96,21	
	TOS-s24≥140 mg/dL (≥7,8 mmol/L)	100	75,3	60	100	0,944 (0,925–0,962)
	TOS-s24>145,4 mg/dL (>8,07 mmol/L)	96,8	82,7	27,9	99,71	0,944 (0,925–0,962)
	HbA_1c_-s24>29 mmol/mol (>4,84%)	67,1	53,3	11,69	94,62	0,642 (0,575–0,709)
	Algoritmo combinado s24	90	83,4	28,42	99,12	

Sb^a^, sensibilidad; Sp^b^, especifidad; VPP^c^, valor predictivo positivo; VPN^d^, Valor predictivo negativo; AUC^e^, Área bajo la curva; IC^f^, intervalo de confianza; TOS-s24^g^, test de O’Sullivan en la semana 24^a^; HbA_1c_ -s24^h^, prueba de la hemoglobina glicosilada en la semana 24^a^; Algoritmo combinado s24^i^, Algoritmo combinado en la semana 24^a^; TOS-s13^j^, test de O’Sullivan en la semana 13^a^; HbA_1c_ -s13^k^, prueba de hemoglobina glicosilada en la semana 13^a^. Algoritmo combinado s13^l^, Algoritmo combinado en la semana 13^a^.

### Análisis de la población sin factores de riesgo

Un total de 1,054 participantes no presentaron factores de riesgo. La prevalencia de la DMG en esta población fue del 1,8%. Los valores de TOS y HbA_1c_ en la semana 24^a^ fueron significativamente más elevados en las participantes que desarrollaron DMG. No se hallaron diferencias en términos de edad (Tabla 2).

La regresión logística no mostró un buen rendimiento diagnóstico, ya que, aunque el análisis clasificó correctamente al 98,4% de las participantes, solo identificó correctamente el 22% de los casos de DMG.

En cuanto a la precisión diagnóstica, el TOS mostró una mayor AUC que la HbA_1c_ (0,953 frente a 0,688, respectivamente) (Figura 1B). El mejor punto de corte para el TOS fue 153,5 mg/dL (8,52 mmol/L) (Sensibilidad: 89,5%; Especifidad: 93,6%; VPP: 20,48%; VPN: 99,79%). Para la HbA_1c_, el mejor punto de corte fue de 28 mmol/mol (4,75%) (Sensibilidad: 77,8%; Especifidad: 52,1%; VPP: 2,87%; VPN: 99,22%) (Tabla 4). Además, se determinaron dos umbrales extremos de HbA_1c_en esta población. El umbral de HbA_1c_ de 25 mmol/mol (4,45%) mostró una sensibilidad similar a la del TOS, por lo que se podría evitar la realización del TOS en las mujeres con valores inferiores a este umbral (sensibilidad 88,9%). Un valor de 37 mmol/mol (5,55%) mostró una especifidad de 98,6% a la hora de diagnosticar la DMG.

### Análisis de la población con factores de riesgo

Un total de 953 participantes presentaban algún factor de riesgo. La prevalencia de la DMG en esta población fue del 10%. Los valores de TOS-s13 y HbA_1c_s13, GCT-s24, y HbA_1c_s24 fueron significativamente superiores en las participantes que desarrollaron DMG. No se observaron diferencias en cuanto a edad. Del mismo modo, las participantes que desarrollaron DMG con frecuencia presentaban un IMC >30 kg/m^2^ y habían desarrollado DMG anteriormente. No hubo diferencias estadísticamente significativas con respecto a la edad >35 años, la macrosomía y los antecedentes familiares (Tablas 2 and 3).

La regresión logística tanto en la semana 13^a^ como en la semana 24^a^ no mostró un buen rendimiento diagnóstico, ya que, aunque el análisis clasificó correctamente al 98,9% de las participantes, solo identificó correctamente el 52% de los casos de DMG.

En cuanto a la precisión diagnóstica en la semana 13^a^, el TOS mostró un AUC de 0,882, siendo el mejor punto de corte 140,5 mg/dL (7,8 mmol/L) (Sensibilidad: 73,1%; Especifidad: 87.7%; VPP: 39,3%; VPN: 96,75%). La HbA_1c_ mostró un AUC de 0,624, siendo el mejor punto de corte 33 mmol/mol (5,15%) (Sensibilidad 51,6%; Especifidad: 67,3%; VPP: 14,74%; VPN: 92,65%) (Figura 1C, Tabla 4). Con respecto a los umbrales extremos, el valor 26 mmol/mol (4,55%) mostró una sensibilidad del 94.5% para descartar DMG, mientras que el valor 39 mmol/mol (5,75%) mostró una especifidad del 98,2% para diagnosticar DMG.

En cuanto a la precisión diagnóstica en la semana 24^a^, el TOS mostró un AUC de 0,944 siendo el mejor punto de corte 145,5 mg/dL (8,08 mmol/L) (Sensibilidad: 96,8%; Especifidad: 82,7%; VPP: 27,90%; VPN: 99,71%). La HbA_1c_ mostró un AUC de 0,642, siendo el mejor punto de corte 29 mmol/mol (4,85%) (Sensibilidad: 67,1%; Especifidad: 53,3%; VPP: 11,69%; VPN: 94,62%) (Figura 1D, Tabla 4). Con respecto a los umbrales extremos, el valor 24 mmol/mol (4,35%) mostró una sensibilidad del 94,7% para descartar DMG, mientras que el valor 39 mmol/mol (5,75%) mostró una especifidad del 98,7% para diagnosticar DMG.

### Validez diagnóstica de los modelos propuestos con datos reales

Con los datos obtenidos, se desarrollaron dos métodos para la detección de DMG: (1) aumentar el punto de corte del TOS con objeto de reducir el número de gestantes que se tienen que someter a la SOG; (2) emplear un algoritmo que combine un punto de corte de HbA_1c_ con buena sensibilidad para descartar DMG, seguido de un SOG con un punto de corte mayor.

#### Gestantes sin factores de riesgo (n=1054)

De acuerdo con la estrategia estándar, todas las gestantes de bajo riesgo tuvieron que someterse al TOS, de las que 187 precisaron una SOG. La prevalencia de DMG en la población con niveles de glucosa en plasma <140 mg/dL (<7,77 mmol/L) (n=866) fue del 0,1% (un caso).

#### Protocolo estándar mejorado (aumentar el punto de corte del TOS a153.5) (Figura 2A)

Con este valor optimizado, solo se tendría que haber realizado la prueba a 83 gestantes. La prevalencia de DMG en la población con niveles de glucosa en plasma <153 mg/dL (<8,49 mmol/L) (n=971) fue del 0,2% (dos casos). Los parámetros estadísticos de este modelo se muestran en la Tabla 4.

**Figura 2:**
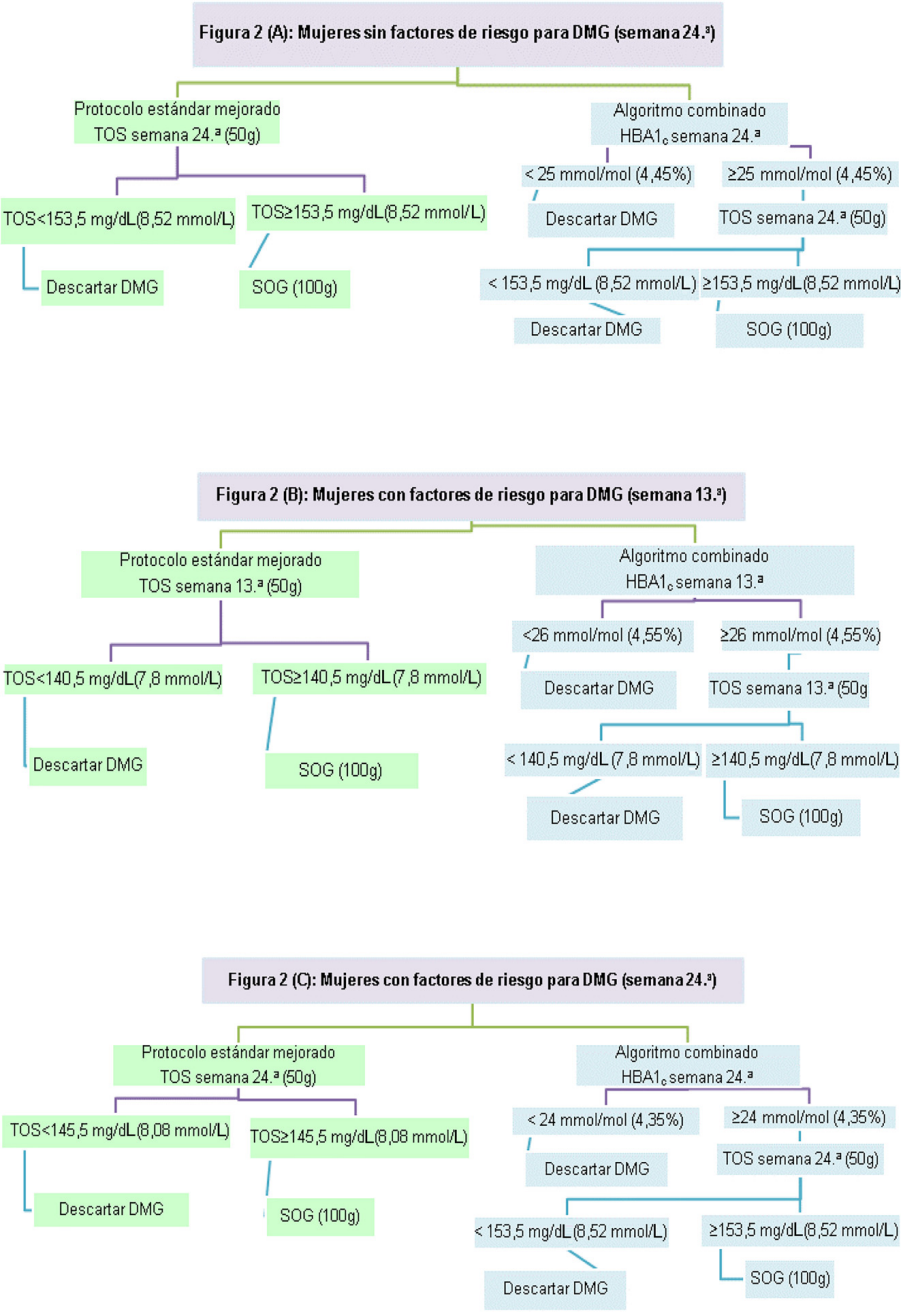
Flujo de trabajo de las dos estrategias para el cribado de DMG. DMG, diabetes mellitus gestacional; TOS-13^a^ semana, test de O’Sullivan en la semana 13^a^; HbA_1c_-13^a^ semana, prueba de la hemoglobina glicosilada en la semana 13^a^; TOS-24^a^ semana, test de O’Sullivan en la semana 24^a^; HbA_1c_-24^a^ semana, prueba de la hemoglobina glicosilada en la semana 24^a^.

#### Algoritmo combinado (Figura 2A)

Teóricamente, si se hubiera aplicado un algoritmo con un umbral de HbA_1c_ de 25 mmol/mol (4,45%) para descartar DMG, habría que haber realizado el TOS a 782 mujeres. Posteriormente, aquellas con un TOS >153,4 (n=61) precisarían la SOG (49 observaciones perdidas de las 1054). En esta cohorte de gestantes, y teniendo en cuenta el diagnóstico final, los parámetros estadísticos del algoritmo fueron: Sensibilidad: 77,8%; Especifidad: 95,2%; VPP: 22,95%, y VPN: 99,57% (Tabla 4).

#### Gestantes con factores de riesgo (n=953) en la semana 13^a^

Con el protocolo clásico, se hubiera realizado el TOS a todas las gestantes con factores de riesgo en la semana 13^a^ (n=953); posteriormente, aplicando el punto de corte estándar (140 mg/dL; 7,77 mmol/L), un total de 188 mujeres precisarían una SOG.

#### Protocolo estándar mejorado (aumentar el punto de corte del TOS a 140,5) (Figura 2B)

Con el nuevo punto de corte, solo se tendría que haber realizado la SOG a 182 mujeres. La prevalencia de la DMG en la población con valores inferiores al mismo fue del 3,2%, por lo que 25 casos no habrían sido diagnosticados. Los parámetros estadísticos de este modelo se muestran en la Tabla 4.

#### Algoritmo combinado (Figura 2B)

Un punto de corte para la HbA_1c_ de 26 mmol/mol (4,55%) permitiría descartar la DMG con una sensibilidad del 94,5%. No obstante, en condiciones reales de uso, aunque nos permitiría descartar DMG en 89 mujeres, cinco se habrían quedado sin diagnosticar (prevalencia de la DMG del 5,6% en la población con un HbA_1c_<26 mmol/mol (4,55%)). Posteriormente, aquellas con un >140.4 (n=151) precisarían una SOG (32 observaciones perdidas de 953). Teniendo en cuenta el diagnóstico final, los parámetros estadísticos de este algoritmo fueron: Sensibilidad: 67,8%, Especifidad: 89,1%, VPP: 40,39%, VPN: 96,21% (Tabla 4).

#### Gestantes con factores de riesgo (n=901) en la semana 24^a^

Con el método tradicional, en la semana 24^a^, se hubieran realizado el TOS todas las participantes con factores de riesgo (n=901); posteriormente, aplicando el punto de corte estándar (140 mg/dL; 7,77 mmol/L), un total de 272 mujeres precisarían una SOG.

#### Protocolo estándar mejorado (aumentar el punto de corte del TOS a 145.5) (Figura 2C)

Un total de 215 mujeres presentaron un TOS >145,4; por lo que, teóricamente, precisarían una SOG. La prevalencia de DMG en esta población fue del 27,9% (60 casos). En el grupo con TOS <145,5, la prevalencia de la DMG fue del 0,3% (solo 2 casos). Los parámetros estadísticos de este modelo se muestran en la Tabla 4.

#### Algoritmo combinado (Figura 2C)

Un punto de corte de 24 mmol/mol (4,35%) para HbA_1c_ permitiría descartar la DMG, con una sensibilidad del 94,7%. Usando datos reales, este valor nos permitiría descartar la DMG en 100 mujeres, pero cuatro hubiesen quedado sin diagnosticar (prevalencia de la DMG del 4% en la población con un HbA_1c_<24 mmol/mol (4,35%)). Posteriormente, 801 mujeres se tendrían que haber sometido al TOS. De estas, 190 mostraron un resultado ≥145,5 y deberían realizarse la SOG con 100 g (23 observaciones perdidas de 901). Teniendo en cuenta el diagnóstico final, los parámetros estadísticos de este algoritmo fueron: Sensibilidad: 90,0%, Especifidad: 83,4%, VPP: 28,42%, VPN: 99,12%.

#### Método del punto de corte extremo para el diagnóstico temprano de DMG en gestantes con riesgo elevado

Si empleáramos un punto de corte con una especifidad teórica para diagnosticar DMG del 98,2%, 39 mmol/mol (5,75%) en nuestra población, se habrían diagnosticado 20 casos, aunque la prevalencia real de la DMG en este subgrupo fue solo del 25% (cinco casos).

## Discusión

Este estudio muestra que el procedimiento estándar (test de O’Sullivan) mejorado con puntos de corte más altos, ofrece la mejor validez diagnóstica para la DMG. La HbA_1c_ podría ser útil con este fin, aunque tiene una validez diagnóstica inferior a la del método de referencia. Aunque la prueba de HbA_1c_ obtuvo un AUC adecuado, esta fue muy inferior a la obtenida con el TOS en todos los grupos estudiados. Esto podría deberse en gran medida a la baja sensibilidad de la HbA_1c_ observada consistentemente en los grupos. La precisión diagnóstica de la prueba de HbA_1c_ para la DMG ha sido analizada recientemente [21], [22], [23], [24]. El valor del AUC en estos estudios oscila entre 0,62 y 0,72. En los mismos, se concluye que la HbA_1c_ no puede sustituir a las pruebas basadas en la sobrecarga de glucosa, aunque podría ser de utilidad como prueba de cribado [23], [ 24] o para identificar a las embarazadas con un riesgo elevado de desarrollar DMG [21]. Además, el papel de la HbA_1c_ en el diagnóstico temprano de la DMG en las mujeres con alto riesgo ha sido analizado en diversos estudios [26], [27], [28]. Con este propósito, en estos trabajos se han empleado puntos de corte extremos que, al maximizar la especifidad, permitirían diagnosticar la DMG en aquellas mujeres que mostraran valores superiores a los mismos. En una revisión sistemática, Kattini et al. [28] concluyeron que un valor límite de entre el 5,7% y el 6,4% identifica a aquellas pacientes que acabarán desarrollando DMG. En nuestro estudio, al aplicar el método de los puntos de corte extremos, obtuvimos varios umbrales de HbA_1c_, tanto para la semana 13^a^ como para la semana 24^a^ de gestación. Teóricamente, estos umbrales podrían diagnosticar o descartar la DMG, maximizando la especifidad o la sensibilidad. El mejor punto de corte para el diagnóstico temprano de la DMG en las pacientes con alto riesgo fue 5,75% (39 mmol/L). Sin embargo, al aplicar el modelo a nuestra población, solo el 25% de los casos con valores superiores a esta cifra desarrollaron DMG. Estos resultados coinciden con los obtenidos por Fong et al. [20], en los que el 27,3% de las embarazadas con un valor de HbA_1c_ superior al 5,7%, evaluadas antes de la semana 20^a^ de gestación desarrollaron DMG. Además, coincide con los resultados de Punnose et al. [29] y Walker et al. [30], quienes concluyeron que la prueba de HbA_1c_ ni es superior a las pruebas basadas en la sobrecarga de glucosa, ni resulta rentable. Por lo tanto, la baja AUC de HbA_1c_ en nuestro estudio, así como en otros [26], [ 29], sugiere que estos puntos de corte extremos podrían no ser adecuados.

De acuerdo con otros autores [31], [32], [33], se propuso un algoritmo combinado. Sin embargo, este método difiere del resto en que se trata de una estrategia en tres pasos donde: (a) en la primera cita con el obstetra, se clasifica a las gestantes (semana 12^a^) como con bajo riesgo o con riesgo elevado, según la presencia o no de factores de riesgo conocidos; (b) se realiza la HbA_1c_ en la semana 13^a^ y/o en la semana 24^a^, según proceda, con el objeto de descartar la DMG; (c) para aquellos casos en los que haya que realizar el TOS, se eleva el umbral de la prueba tanto para las gestantes con bajo riesgo como para aquellas con un riesgo elevado, con el fin de evitar la realización innecesaria de la SOG. Sin embargo, los algoritmos resultantes mostraron un rendimiento diagnóstico inferior que el protocolo estándar mejorado (Tabla 4).

Al elaborar este artículo, la pandemia de COVID-19 se ha convertido en el mayor problema de salud pública de las últimas décadas. Con el fin de evitar la posible exposición de las gestantes al virus, autoridades sanitarias y sociedades científicas, han propuesto nuevos protocolos para el diagnóstico de DMG basados en la HbA1_c_ y la glucosa en plasma en ayunas o aleatoria [34], [35], [36], [37]. La eficacia de estos métodos a para detectar DMG y sus posibles complicaciones en el embarazo se han estudiado exhaustivamente a partir de datos del “Estudio sobre la Hiperglucemia y los Resultados Adversos del Embarazo” (HAPO) [38]. Este estudio muestra que los métodos basados en la prueba de HbA_1c_ y/o de glucosa en plasma en ayunas [34], [ 35] están asociados con un aumento en el número de gestantes con DMG no diagnosticada, con índices significativamente mayores de complicaciones en el embarazo. Sin embargo, el método basado en los niveles de glucosa en ayunas, seguido de una prueba de SOG selectiva [36], aunque asociado con un mayor número de DMG no diagnosticada, no está unido a una mayor incidencia de resultados adversos. Por todo ello, los autores recomiendan que se evalúen los riesgos y beneficios de cada propuesta, en el contexto actual de pandemia.

En este estudio se demuestra que se debería reconsiderar en todos los grupos el punto de corte estándar del TOS (140 mg/dL: 7,77 mmol/L). Para las gestantes de bajo riesgo, el nuevo valor es especialmente relevante (153,5 mg/dL: 8,52 mmol/L). Aumentar la especifidad podría implicar menor sensibilidad de la prueba. No obstante, este punto de corte mostró una sensibilidad del 89,5% y, en la práctica real, los casos sin diagnosticar aumentarían ligeramente del 0,1% al 0,2%. En el contexto de bajos índices de prevalencia comunitarios, como es el caso de nuestra cohorte, se ha propuesto ajustar el punto de corte del TOS a 145 mg/dL (8,05 mmol/L) en embarazos gemelares para evitar falsos positivos de DMG [39]. Además, la estrategia mejorada podría ser costo-eficiente. En la población de bajo riesgo, se podrían haber evitado el 55,6% de los TOS. En la población de alto riesgo en la semana 13^a^ solo se habría evitado realizar el 3,2% de las SOG. Sin embargo, en la semana 24^a^, la ventaja fue evidente, pudiéndose haber evitado el 21% de las SOG.

### Puntos fuertes y limitaciones del estudio

Este estudio se ha realizado con una de las mayores cohortes publicadas hasta la fecha. Su principal limitación radica en que en España se aplica un método diferente al recomendado por la ADA o la OMS para diagnosticar DMG, lo que limita en parte la extrapolación de los resultados obtenidos. Otras limitaciones son que el presente estudio es unicéntrico, con una prevalencia de DMG del 5,7% y las gestaciones múltiples no se excluyeron (prevalencia <1,5%). Aunque hubo un 9,7% de pérdidas durante el seguimiento, es un porcentaje aceptable que no pone en riesgo la validez de los resultados obtenidos.

## Conclusiones

Este estudio muestra que se puede optimizar el cribado de DMG aplicando nuevos puntos de corte para el O’Sullivan. Además, se muestra que, en términos de precisión diagnóstica, la HbA_1c_ por sí misma o en un algoritmo combinado, es inferior a la del método mejorado del O’Sullivan. Por otra parte, el método del punto de corte extremo para el diagnóstico temprano de la DMG en la población de alto riesgo basado en la HbA_1c_ resulta menos eficiente que la estrategia estándar o la mejorada propuesta en este estudio.

Es necesario realizar más estudios sobre los puntos de corte del test de O’Sullivan para poder aplicar cambios reales al protocolo de cribado de DMG.

## Supplementary Material

Supplementary Material

## Material Suplementario

La versión en línea de este artículo ofrece material suplementario (https://doi.org/10.1515/almed-2020-0118).
