# Supplementary material for: Mejora del protocolo de cribado de diabetes gestacional: estudio de validación diagnóstica
Source: Adv Lab Med. 2021 Feb 11;2(1):97–107. [Article in Spanish] doi: 10.1515/almed-2020-0118 (PMC10197422; doi:10.1515/almed-2020-0118)
Supplement: Supplementary file 1 — Supplementary Material [file j_almed-2020-0118_suppl.pdf]

Material Suplementario

Procedimientos analíticos

Para la prueba de HbA_{1c}, se recogieron muestras de sangre venosa en tubos EDTA y se realizaron las mediciones mediante cromatografía líquida de alta eficacia (HPLC) en el instrumento Variant II TURBO (Bio-rad, Hercules, California). Este sistema está certificado por el Programa Nacional de Estandarización de la Glicohemoglobina (NGSP) y descrito en el "Ensayo de control de la diabetes y de sus complicaciones" (DCCT).

Para evitar sesgos preanalíticos relacionados con la glicólisis, para las pruebas de glucosa, se recogieron muestras de sangre venosa en tubos con fluoruro de sodio como inhibidor de la glucólisis [16]. Para el test de O´Sullivan, se tomaron muestras de sangre venosa una hora después de la administración oral de 50g de glucosa. Para la SOG, se tomaron muestras de sangre venosa basalmente y una, dos, y tres horas después de la administración oral de 100g de glucosa. La glucosa en plasma se midió mediante el método de glucosa oxidasa en un analizador modular Cobas 8000 (Roche Diagnostic, Basel, Suiza).

La DG se diagnosticó de acuerdo con los cristerios del GEDE [10] y el Grupo Nacional de DAtos sobre la Diabetes (NDDG) [11].

Análisis estadístico

La normalidad en la distribución de los datos se evaluó con la prueba de Kolmogorov-Smirnov. Para la estradística descriptiva, se calcularon la media, mediana, desviación estándar y rango intercuartílico de las variables continuas (edad, HbA_{1c}, GCT) y las frecuencias y porcentajes de las variables discretas (cadca factor de riesgo para la DMG).

Se aplicó el test U de Mann-Whitney para evaluar las diferencias entre las variables continuas. Para las diferencias entre las variables discretas, se empleó el test chi cuadrado.

Los resultados se expresaron en odds ratios (OR), intervalos de confianza del 95% (IC) y media ± desviación estándar. Un valor de *p* de <0.05 se consideró estadísticamente significativo.

Finalmente, se realizó un análisis de regresión logística multivariante de todas las variables.

Para determinar la validez diagnóstica de las pruebas de HbA_{1c} y TOS, se calcularon su sensibilidad, especifidad, valor predictivo positivo (VPP), valor predictivo negativo (VPN), la curva ROC, y el área bajo la cuerva (AUC). Además, con el fin de maximizar la especifidad (para detectar DMG) y la sensibilidad (para descartar DMG), se realizó un análisis de valores extremos. El análisis estadístico se realizó en toda la población del estudio, así como en los subgrupos de embarazadas con y sin factores de riesgo. Todos los análisis estadísticos se realizaron con el programa SPSS v15 (SPSS Inc., Chicago, Illinois).

Estrategias para el cribado de la DMG

En una segunda fase del estudio, con los datos obtenidos se desarrollaron dos estrategias para el cribado de la DMG: estrategia 1) aumentar el punto de corte en la prueba de cribado para reducir el número de mujeres que se someten a la SOG; estrategia 2) usar un algoritmo que combine puntos de corte extremos de HbA_{1c} con sensibilidad suficiente para descartar la diabetes, seguido del uso de un punto de corte mayor para el TOS. Al emplear estas diferentes estrategias en nuestra cohorte, y teniendo en cuenta el diagnóstico final, se pudo comprobar qué aproximación era la más apropiada. Es decir, aquella con al menos la misma validez diagnóstica que la estrategia convencional, pero con menos inconvenientes. Además, se aplicó el método del punto de corte extremo, que maximiza la especifidad para diagnosticar con seguridad la DMG, al grupo de gestantes de alto riesgo en el primer trimestre de embarazo.
